# Poor Lower Extremity Functioning Is Associated with Modest Increased Incidence of Probable Dementia

**DOI:** 10.3390/geriatrics6030077

**Published:** 2021-08-10

**Authors:** Sergio L. Teruya, Cara Dimino, Kevin D. Silverman, Thelma Mielenz

**Affiliations:** Department of Epidemiology, Columbia University Mailman School of Public Health, 722 West 168th Street, New York, NY 10032, USA; c.r.dimino@gmail.com (C.D.); kdsilverman19@gmail.com (K.D.S.); tjm2141@cumc.columbia.edu (T.M.)

**Keywords:** aging, physical functioning, Short Physical Performance Battery, cognitive function

## Abstract

Lower extremity functioning in older adults provides a measure of poor physical performance and can predict negative health outcomes. The consequences of reduced lower extremity functioning on cognitive decline, measured as time-varying variables, have not been well documented in previous studies. We aimed to evaluate whether lower extremity functioning is associated with an increased incidence rate of probable dementia among older adults using data from the National Health and Aging Trends Study (NHATS). Participants (*n* = 6457) were followed for 8 years to examine the relationship between lower extremity functioning, as measured by the Short Physical Performance Battery (SPPB), and incident probable dementia. Using weighted data, a multivariable Poisson regression with generalized estimating equations (GEE) was used to calculate incidence rate ratios (IRR), adjusting for covariates and clustering. Participants with low SPPB scores (0–5) had a 5% increase in incident probable dementia when compared with those who had good SPPB scores (10–12) in the adjusted model (IRR = 1.05; 95% CI = 1.04–1.07). Lower extremity functioning is associated with a modest increase in incident probable dementia. The SPPB score may be helpful in identifying subjects at risk of dementia. Efforts aimed at improving physical functioning may lead to better cognitive outcomes.

## 1. Introduction

By 2050, one in six people in the world will be over age 65 (16%), and the population aged 80 or older is expected to triple [1]. This aging population underlines the importance of maintaining physical and cognitive functioning later in life. Evidence suggests that worsening cognitive function is correlated with a decline in physical performance and gait (specifically in subjects with dementia) [2], and cardiovascular fitness is protective against cognitive decline among older adults [3,4]. Among older adults, maintaining a higher level of cardiorespiratory fitness may help in mitigating cognitive impairments [5,6,7]. Among people not involved in cognitive activities (e.g., reading, playing games, or playing musical instruments), engaging in high levels of physical activity (PA), which is associated with increased physical performance, reduces the odds of cognitive impairment [8].

Previous studies suggest that physical performance is predictive of cognitive performance across all cognitive domains [9]. Gender is a moderator in the association between PA and cognition, with the effect size for improvement being greater among women [10,11], and performing and sustaining any type of PA can result in improved physical function (PF).

Current literature has established an association between worsening physical performance and decline in cognitive function [2,9,12,13,14]. The association between lower extremity functioning and cognitive function, and the dynamic assessment of cognitive status (i.e., changes over time) has yet to be explored. The purpose of this study was to determine whether poor lower extremity functioning is associated with an increased rate of cognitive decline, and to explore whether being female is protective against poorer cognition. The study was based on data collected by the National Health and Aging Trends Study (NHATS), a large nationally representative cohort. Our hypothesis was that poor lower extremity functioning is associated with an increased incidence rate of probable dementia over an 8-year follow-up period among older adults in the U.S. population.

## 2. Materials and Methods

Data used in this study were from the National Health and Aging Trends Study (NHATS), Rounds 1 through 8 (2011–2018) [15]. NHATS collects information about characteristics and functioning of older adults.

NHATS data are gathered in persons from a nationally representative sample of 35.3 million Medicare beneficiaries aged 65 and older who reside in the United States. Data acquisition started with an initial 2011 sample. Follow-up interviews and assessments (rounds) were performed annually, and the cohort was replenished in 2015. Information collected by NHATS includes physical and cognitive function tests.

NHATS is funded by the National Institutes on Aging in collaboration with Johns Hopkins University Bloomberg School of Public Health, with data collection by Westat.

### Study Population

**Figure 1:**
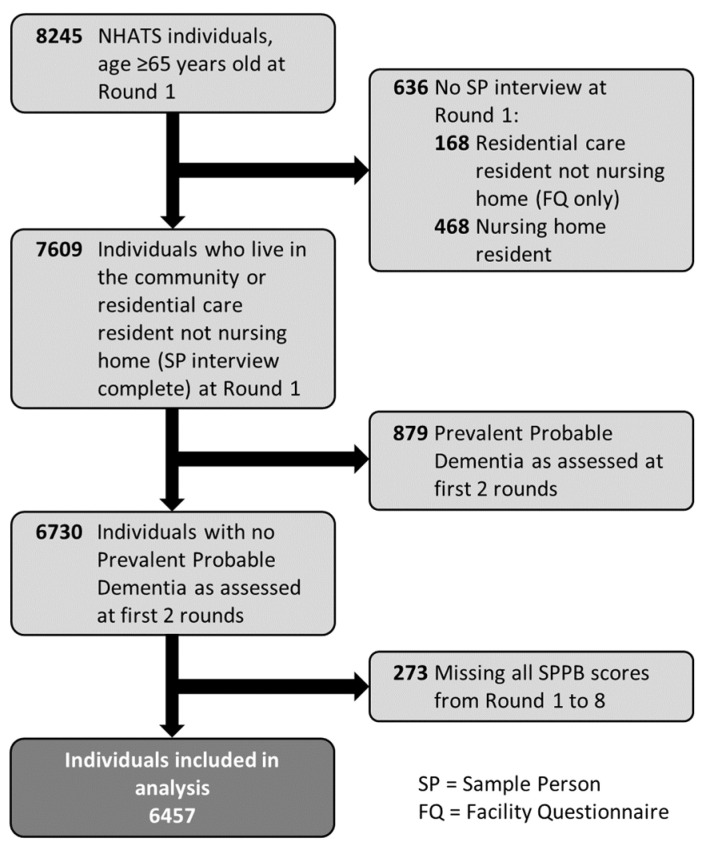
Flow diagram of study participant inclusion.

A total of 8245 individuals aged 65 and older were enrolled in NHATS at Round 1 (2011), with follow-up data collection occurring annually thereafter. At Round 1, subjects living in residential care facilities (*n* = 168) and nursing homes (*n* = 468) where physical and cognitive assessments could not be performed were excluded from analysis. Of the 7609 individuals living in the community or residential care residents able to complete the physical and cognitive assessments, 879 were excluded due to presenting with probable dementia at Rounds 1 and 2. An additional 273 participants missing all SPPB scores from Round 1 through Round 8 were removed from the remaining group of 6730 individuals with possible dementia or no dementia at baseline. The final study population consisted of 6457 individuals (Figure 1).

Diagnosis of incident probable dementia was defined by previous NHATS studies [16]. Classification of NHATS participants’ dementia status (probable dementia, possible dementia, or no dementia) was determined by three types of collected information: report of diagnosis by a physician, AD8 Dementia Screening Interview, and cognitive tests [17]. The NHATS definition, as measured by Kasper et al. 2013, demonstrates strong sensitivity and specificity against a dementia diagnosis [17].

A report of diagnosis by either the NHATS participant or a proxy that the study participant was told by a physician that he/she had dementia or Alzheimer’s disease classified the person as having probable dementia. The AD8 Dementia Screening Interview [18,19] is an 8-item instrument administered to proxy respondents who are answering the interview for the participant. It assesses memory, temporal orientation, judgment, and function. Five cognitive tests assess 3 domains: memory (immediate and delayed 10-word recall), orientation (date, month, year, and day of the week; naming the President and Vice President), and executive function (clock drawing test). Scores range from 0 through 20 for memory, 0 through 8 for orientation, and 0 through 5 for executive functioning.

Participants with proxy respondents not reporting a diagnosis of dementia who gave answers to the AD8 with a score of 2 or higher were also classified as having probable dementia. Cognitive measures were used for determination of dementia status in subjects not classified by the first two criteria. Impairment in a cognitive domain was defined as a score at or below 1.5 standard deviations (SD) from the mean. According to this definition, impairment for the 3 domains was defined as follows: score ≤ 3 for orientation, score ≤ 3 for memory, and score ≤ 1 for executive functioning. Probable dementia was defined as impairment in at least 2 cognitive domains, and possible dementia as impairment in 1 domain.

Due to potential learning effects of the cognitive tests with successive evaluations, a two-round criteria was imposed for the diagnosis of prevalent and incident probable dementia [16]. For prevalent dementia, individuals had to either have been diagnosed with probable dementia for two consecutive rounds or diagnosed with probable dementia in one round before death or loss to follow-up. For incident dementia, previously not demented individuals had to either present with probable dementia for two consecutive rounds or have probable dementia in one round followed by death or loss to follow-up.

We used a physical performance measure called the Short Physical Performance Battery (SPPB) developed by Guralnik et al. (1994), which is a performance measure that tests the functional ability to perform lower extremity functioning. Poor performance on the tests used in the SPPB are associated with lower quality of life [20,21] and are predictive of adverse health outcomes in older adults, including hospitalization, nursing home admission, disability, and all-cause mortality [22,23,24,25,26,27]. The original SPPB consists of three balance, walking speed, and repeated chair stand tests. The balance tests assess the ability to stand with feet in side-by-side, semi-tandem, and tandem positions for about 10 s. The gait speed test evaluates normal walking speed while covering a course of 3 m. The chair stand test measures the time needed to stand up from a chair five times as quickly as possible without using the arms. The overall score is obtained by adding up the scores of each test score, ranging from 0 through 4. Total scores were categorized as poor (0–5), fair (6–9), and good (10–12), consistent with previous studies utilizing the SPPB [28].

Scoring for the balance tests was categorized as follows: 0 (not attempted, did not complete side-by-side stand); 1 (completed side-by-side stand and did not complete or did not attempt semi-tandem stand); 2 (completed semi-tandem and held full tandem for 0 to 2.99 s or did not attempt full tandem); 3 (completed semi-tandem and held full tandem for 3 to 9.99 s; and 4 (completed full tandem). Scoring for walking speed was categorized as follows: 0 (not attempted or attempted but not completed); 1 (≤0.441 m/s); 2 (0.442–0.624 m/s); 3 (0.625–0.798 m/s); and 4 (≥0.799 m/s). Finally, the scoring for the repeated chair stands was categorized as follows: 0 (not attempted or attempted but not completed); 1 (≥16.70 s); 2 (13.70–16.69 s); 3 (11.20–13.69 s); and 4 (≤11.19 s).

Both walking speed and the repeated chair stand score categories were based on the original SPPB’s quartiles [26]. Further details on scoring criteria are available through NHATS [29].

Factors associated with PF and/or cognitive decline were selected as independent variables for multivariate analyses. These included age range, gender, race/ethnicity, education, comorbidities, and obesity. This information was acquired at baseline (Round 1).

Age was categorized into 65–69, 70–74, 75–79, 80–84, 85–89, and ≥90. Gender included men and women. Race/ethnicity was categorized as non-Hispanic white, non-Hispanic black, non-Hispanic other (included American Indian, Asian, Native Hawaiian and Pacific Islander), or Hispanic. Prevalence and incidence rates of dementia have been found to be higher among both Hispanics and non-Hispanics blacks as compared to non-Hispanic whites [30,31]. Level of education was categorized into ≤8th grade; 9th–12th grade (no diploma); high school graduate (high school diploma or equivalent); or higher. Glymour et al. reported that increasing quality and level of education is associated with improvement in memory tests in late adulthood [32]. Langa et al. also reported a decline in the prevalence of dementia in recent years associated with an increase in educational attainment [33]. Body mass index (BMI) was calculated from self-reported height and weight and categorized as follows: <18.5 (underweight), 18.5–24.9 (normal), 25–29.9 (overweight), and ≥30 (obesity). Depressive symptoms were measured with the two-item Patient Health Questionnaire-2 (PHQ-2) [34] which captures cognitive/affective symptoms of anhedonia and depressed mood by asking “Over the last month, how often have you/has the sample person (a) had little interest or pleasure in doing things, and (b) felt down, depressed, or hopeless?”. Responses were based on a 4-point scale. The combined score was used as an overall symptom severity score, and a score > 3 was used to indicate probable major depression (PMD) [34]. Additional comorbidities included the following chronic conditions reported by the participant: heart attack, heart disease, hypertension, osteoarthritis, osteoporosis, diabetes, lung disease, stroke, and cancer. A comorbidity scale was based on the number of conditions reported (from 0 to 4+). It has been suggested that improvements in prevention and treatment of these comorbid medical conditions are associated with a reduction in the incidence of dementia [33]. Freedman et al., using data from the first five rounds of NHATS, found a positive association between prevalence of dementia and history of heart attack, heart disease, hypertension, diabetes, stroke, and obesity. They also reported a decline in incidence of dementia among those with no history of vascular conditions and risk factors [16].

Initial descriptive analyses of demographic characteristics and dementia classification by baseline SPPB score were examined with chi-squared tests. To evaluate the association of SPPB score as a three-level ordinal variable (i.e., poor, fair, and good) as time-varying and the incidence rate of probable dementia, a generalized estimating equation (GEE) analysis was employed, specifying the log link function with a Poisson distribution [35,36,37]. A GEE analysis accounted for data that were correlated with our repeated measures from Round 1 through Round 8, and for subjects whose cognitive status changed back to no dementia after being classified with probable dementia at a previous round [35,36].

The adjusted model included time-dependent covariates (age range, comorbidity, and obesity status) [38]. Age group, comorbidities, and BMI were assessed for linearity with dementia classification, and they were included in the models as ordinal variables. Remaining covariates (i.e., gender, race/ethnicity, and education) were modeled as time-invariant variables. Clustering was accounted for in unadjusted and adjusted models. To estimate the rate ratios and confidence intervals (CIs), a log link and exchangeable working correlation matrix were specified in the models [39]. Robust SEs for the parameter estimates were used to control for mild violation of the distribution assumption [37]. All statistical analyses were performed using SAS University Edition Version 3.8 (SAS Institute Inc., Cary, NC, USA), and the final models were run in STATA Version 16.1 (StataCorp LLC, College Station, TX, USA).

We used the Round 8 (2018) replicate weights developed by NHATS to produce nationally representative estimates of the older Medicare population [40]. These weights used the modified balance repeated replication method to adjust for variance estimates. All means, proportions, and measures of association are based on the weighted data, and sample sizes are unweighted [35].

## 3. Results

The study cohort consisted of 6457 NHATS participants without probable dementia at the first two rounds for which SPPB scores were available for at least one of the eight years of follow-up. Out of the total analyzed sample, 578 subjects had missing SPPB at baseline (Round 1), and therefore only 5879 provided data to characterize the study population at the initial round (Table 1). After accounting for the NHATS-provided study weights, 17.1% of study subjects had a poor SPPB score at baseline, while 29.4% and 53.6% had fair and good SPPB scores, respectively. Of all study participants, 1.7% were found to have probable dementia at baseline. Among subjects with poor physical performance scores, 4.2% were diagnosed with probable dementia at baseline, compared to 0.6% among those with good physical performance scores (*p*-value < 0.0001). The covariates included in the analysis were all significantly associated with SPPB score (*p*-value < 0.0001) in the univariate analysis (chi-squared tests). Of all study participants with no prevalent probable dementia at baseline, 11% progressed to incident probable dementia and 89% remained with no incident probable dementia at the end of the observation period. Only seven subjects improved and were no longer categorized as incident probable dementia (Table 2).

In the unadjusted Poisson regression model (Table 3), subjects with poor SPPB scores (0–5) had an 8% increase in the incidence rate of probable dementia compared to those with good SPPB scores (10–12) (IRR = 1.08; 95% CI = 1.07–1.10). The magnitude of the association decreased when comparing the incidence rate of probable dementia between individuals with fair SPPB score (6–9) to those with good SPPB scores (IRR = 1.02; 95% CI = 1.01–1.02). Adjusting for age, gender, race, education, BMI, PMD, comorbidities, and clustering slightly reduced the magnitude of the relationship between SPPB score and the incidence rate of probable dementia. The incidence rate of probable dementia in subjects with poor SPPB scores was 1.05 times that of subjects with good SPPB scores (IRR = 1.05; 95% CI = 1.04–1.07). The incidence rate ratio comparing subjects with fair scores to those with good scores was 1.01, but the association was insignificant (*p*-value > 0.05). Similar results were obtained after stratification by gender, across levels of SPPB score in both the crude and adjusted models (data not shown). However, the incidence rate of probable dementia among men with fair SPPB scores was significantly higher than that of men with a good SPPB score in the adjusted model (*p*-value = 0.02). The same association was not significant among women (*p*-value = 0.70).

## 4. Discussion

This study investigated whether lower extremity functioning, when treated as a time-dependent variable, is associated with cognitive decline. Our findings show that there is a significant association between SPPB score and incident probable dementia. We found that participants with poor and fair SPPB scores demonstrated increased incident probable dementia when compared with participants with good SPPB scores in the unadjusted model. We found that stratification by gender did not result in a significant difference in probable dementia between SPPB scores.

Our findings are consistent with previous studies finding an association between worsening PF and cognitive decline [2,9,12,13,14]. Unlike previous studies, this approach involved the analysis of repeated observations accounting for possible variations in the dementia classification over the 8-year period. Our data show that most study participants without prevalent probable dementia at baseline remained without probable dementia during the study, while a small proportion progressed to incident probable dementia, and few individuals improved their cognitive status after being classified as incident probable dementia. These possible variations in the participants’ cognitive status were considered in estimating the measures of association in our analysis.

Gender as an effect modifier was examined in the study. Overall, gender was not associated with a significant change in the association between SPPB score and incident probable dementia. The association only became non-significant when comparing women with a fair SPPB score to those with a good SPPB score. In previous studies, gender was found to be an important moderator of the relationship between PA and cognition, with a larger effect size for executive functions found among women [10,11]. In those studies, PA was measured by engagement in different types of training (e.g., aerobic, resistant, and multimodal). Since performing and sustaining any type of PA can result in improved PF [41] and thus better SPPB scores, we expected to find a similar effect modification by gender in this study. Given that stratification by gender produced similar point estimates and confidence intervals with measures of association close to the null, particularly when comparing those with fair SPPB scores to those with good SPPB scores, the clinical significance is minimal and we cannot determine the role of gender in the relationship between lower extremity and cognitive function. This discrepancy between our findings and those of previous studies may be due to PF only being a proxy for PA. While measuring PF with performance-based measures (e.g., SPPB) is standard in epidemiologic studies, PA is less consistently defined. This may account for the different results by gender stratification found.

Our study has several strengths. Firstly, it is based on eight years of observational data. This allowed us to implement the two-round criteria of incident probable dementia more easily. Studying incident probable dementia as a dynamic outcome, which may progress over time, was a novel approach. Finally, the two-round criteria imposed to define the incidence of probable dementia ensured that we recorded actual longer-lasting and more definitive cases of probable dementia, and not transient changes in mental status due to acute decompensating conditions.

This study had several limitations. While there is a significant relationship between poor SPPB performance and incident dementia, it is possible that this relationship is bidirectional. Those with dementia may be more likely to have difficulty participating in the SPPB, making the directionality of the relationship difficult to determine. In a recent study looking at the prevalence of probable dementia using NHATS data [16], a report of a diagnosis of dementia by the study subject or proxy represented more than half of the total number of cases. The AD8 screening interview identified a smaller proportion of cases. Thus, the more clinical and scientific cognitive function tests did not contribute as much to the diagnosis of probable dementia. Self-report and proxy questionnaires are prone to misdiagnosis as compared to more objective cognitive tests. In most epidemiological studies, diagnosis criteria based on the Diagnostic and Statistical Manual of Mental Disorders (DSM), currently in its fifth edition, are employed to identify cases of dementia [42]. Further, it is also possible that the subset of subjects who were classified as having probable dementia in one round followed by death or loss to follow-up actually represented ill subjects facing overall declining health, and not cases of new probable dementia. This could cause a differential misclassification of the outcome, falsely inflating the number of cases of probable dementia among individuals with poor SPPB scores and biasing our results away from the null. However, only a small proportion of subjects were lost to follow-up in the study. Lastly, NHATS is a large study with many participants. Thus, it is possible that the SPPB and cognitive testing may not have been performed consistently across all sites and participants.

PF is a modifiable determinant of aging well in older individuals and may be improved with exercise training and PA. A systematic review and meta-analysis of 48 studies among adults aged 60 and older suggested that exercise training significantly improves both physical and cognitive function, and that exercise-induced improvements in PF are positively correlated with cognitive function [43]. This study supports the hypothesis that poor lower extremity functioning is associated with an increased incidence rate of probable dementia among older adults in the U.S., and this relationship did not differ by gender. Annual administration of the SPPB could identity older adults at risk of probable dementia, making it possible to intervene through evidence-based PA and exercise programs to decrease the burden of dementia.

## Figures and Tables

**Table 1:** Characteristics of Medicare beneficiaries by SPPB total score at baseline, NHATS, 2011 ^1^.

Variables, % (*n*) ^2^	Total	Total SPPB SCORE		
		Poor (0–5)	Fair (6–9)	Good (10–12)
Total	(5879)	22.4 (1314)	32.5 (1913)	45.1 (2652)
Dementia classification				
Possible or no dementia	98.3	95.8	97.9	99.4
Probable dementia	1.7	4.2	2.1	0.6
Age group, yr				
65–69	30.2	14.5	20.8	40.3
70–74	26.5	18.4	25.8	29.5
75–79	19.2	18.0	22.5	17.8
80–84	13.8	20.5	18.6	9.0
85–89	7.2	17.4	9.4	2.8
90+	3.1	11.1	2.9	0.6
Gender				
Women	56.0	67.0	61.6	49.4
Men	44.0	33.0	38.4	50.6
Race				
White, non-Hispanic	82.9	77.2	79.1	86.8
Black, non-Hispanic	7.4	11.5	9.7	4.9
Hispanic	6.5	8.8	8.1	4.8
Other	3.2	2.5	3.2	3.4
Education				
≤8th grade	8.4	15.6	10.6	4.8
9th–12th grade (no diploma)	10.7	14.6	13.3	7.9
High school graduate or higher	81.0	69.7	76.1	87.2
BMI				
<18.5 (underweight)	1.8	3.2	1.8	1.4
18.5–24.9 (normal)	31.1	31.4	29.1	32.1
25.0–29.9 (overweight)	38.9	32.7	36.5	42.1
≥30.0 (obesity)	28.2	32.7	32.6	24.3
Depressive symptoms				
No probable major depression	94.9	86.9	95.1	97.3
Probable major depression	5.1	13.1	4.9	2.7
Comorbidity scale				
0	19.4	8.5	14.6	25.6
1	32.1	24.0	29.1	36.3
2	26.7	29.0	30.5	23.9
3	13.7	20.5	15.5	10.6
4 or more	8.1	18.0	10.3	3.7

Note. SPPB = Short Physical Performance Battery; NHATS = National Health and Aging Trends Study; BMI = body mass index.^1^ Analyses of weighted data from NHATS. Sample totals provided along with weight percentages. ^2^ *p*-value < 0.0001 for chi-square tests for all categorical variables.

**Table 2:** Changes in dementia classification at the end of the observation period, NHATS, 2011–2018 ^1^.

Classification Change	*n* (%)
Progressed to probable dementia	729 (11.3)
Remained with no probable dementia ^2^	5721 (88.6)
Improved after being classified as probable dementia	7 (0.1)
Total	6457 (100.0)

Note. NHATS = National Health and Aging Trends Study. ^1^ Analyses of weighted data from NHATS. ^2^ No probable dementia included subjects with either possible dementia or no dementia.

**Table 3:** Total SPPB score and incidence rate of probable dementia, NHATS, 2011–2018 ^1^.

Total SPPB Score	Unadjusted Rate Ratio (95% CI)	Adjusted Rate Ratio (95% CI) ^2^
0–5	1.08 (1.07–1.10) *	1.05 (1.04–1.07) *
6–9	1.02 (1.01–1.02) *	1.01 (1.00–1.01)
10–12	Reference	Reference

Note. SPPB = Short Physical Performance Battery; NHATS = National Health and Aging Trends Study; CI = confidence interval. ^1^ Analyses of weighted data from NHATS. ^2^ Multivariable Poisson regression generalized estimating equation (GEE) model adjusted for age, gender, race, education, body mass index (BMI), probable major depression (PMD), and comorbidities accounting for clustering. * *p*-value < 0.001.

## Data Availability

NHATS uses publicly available data.
